# Data-Driven Prediction of Fatigue in Parkinson’s Disease Patients

**DOI:** 10.3389/frai.2021.678678

**Published:** 2021-09-13

**Authors:** Dong Goo Lee, Adrian Lindsay, Adam Yu, Samantha Neilson, Kristen Sundvick, Ella Golz, Liam Foulger, Maryam Mirian, Silke Appel-Cresswell

**Affiliations:** ^1^ Faculty of Medicine, University of British Columbia, Vancouver, BC, Canada; ^2^ Pacific Parkinson’s Research Centre, Djavad Mowafaghian Centre for Brain Health, University of British Columbia, Vancouver, BC, Canada; ^3^ Division of Neurology, Djavad Mowafaghian Centre for Brain Health, University of British Columbia, Vancouver, BC, Canada

**Keywords:** Parkinson’s disease, fatigue, machine learning, artificial intelligence, sex, random forest, Boruta, principal components analysis

## Abstract

**Introduction:** Numerous non-motor symptoms are associated with Parkinson’s disease (PD) including fatigue. The challenge in the clinic is to detect relevant non-motor symptoms while keeping patient-burden of questionnaires low and to take potential subgroups such as sex differences into account. The Fatigue Severity Scale (FSS) effectively detects clinically significant fatigue in PD patients. Machine learning techniques can determine which FSS items best predict clinically significant fatigue yet the choice of technique is crucial as it determines the stability of results.

**Methods:** 182 records of PD patients were analyzed with two machine learning algorithms: random forest (RF) and Boruta. RF and Boruta calculated feature importance scores, which measured how much impact an FSS item had in predicting clinically significant fatigue. Items with the highest feature importance scores were the best predictors. Principal components analysis (PCA) grouped highly related FSS items together.

**Results:** RF, Boruta and PCA demonstrated that items 8 (“Fatigue is among my three most disabling symptoms”) and 9 (“Fatigue interferes with my work, family or social life”) were the most important predictors. Item 5 (“Fatigue causes frequent problems for me”) was an important predictor for females, and item 6 (“My fatigue prevents sustained physical functioning”) was important for males. Feature importance scores’ standard deviations were large for RF (14–66%) but small for Boruta (0–5%).

**Conclusion:** The clinically most informative questions may be how disabling fatigue is compared to other symptoms and interference with work, family and friends. There may be some sex-related differences with frequency of fatigue-related complaints in females and endurance-related complaints in males yielding significant information. Boruta but not RF yielded stable results and might be a better tool to determine the most relevant components of abbreviated questionnaires. Further research in this area would be beneficial in order to replicate these findings with other machine learning algorithms, and using a more representative sample of PD patients.

## Introduction

Parkinson’s disease (PD) is a neurodegenerative disorder that affects over 6 million patients worldwide, as well as their families and caregivers (GBD 2016 Neurology Collaborators, 2019). PD features numerous motor and non-motor manifestations across a diverse patient population (Clarke, 2007). Among these symptoms, the prevalence of fatigue has been reported to affect upto 81% of PD patients, and approximately one-third of PD patients consider fatigue to be their most disabling symptom (Stocchi et al., 2014).

However, there is a lack of a universally accepted definition for fatigue, and multiple components of fatigue have been discussed in the research literature (Kostić et al., 2016). For example, a subjective component of fatigue involves a sense of exhaustion, weakness and lack of energy, while an objective component of fatigue involves an impaired ability to initiate and sustain voluntary actions (Kostić et al., 2016).

Different measures of fatigue show varying degrees of emphasis on assessing each component of fatigue. Consequently, previous researchers have recognized the difficulties associated with identifying the appropriate method of measuring fatigue, and evaluated fatigue rating measurement scales to improve the recognition and treatment of fatigue in PD patients. For instance, the Movement Disorders Society Task Force on Rating Scales for PD reviewed the descriptive properties, psychometric performance, and the overall impression of seven fatigue rating scales which have been used to assess PD patients (Friedman et al., 2010).

Amongst the scales that were assessed, the Fatigue Severity Scale (FSS) received the highest evaluation (Friedman et al., 2010). The FSS is a unidimensional self-administered 9-item survey, which requires the respondent to score from 1 (strongly disagree) to 7 (strongly agree) as their degree of agreement regarding each of the nine statements, such as “I am easily fatigued” (Krupp et al., 1989). The decision to use the FSS is up to the discretion of the clinician, and it is not used in all patients with PD. Once the respondent has answered all of the questions, the scores for all items are summed up. If the sum is equal to or greater than the threshold score of 36, the patient is determined to have clinically significant fatigue (Friedman et al., 2010). The FSS received the highest evaluation of “recommended” for both screening and severity rating (Friedman et al., 2010). This means that the FSS has been used in clinical studies involving PD and other diseased populations, and has been found to be a psychometrically valid and reliable measure (Friedman et al., 2010). In contrast, scales other than the FSS were assessed to be ‘‘suggested’’ scales, which failed to meet all the criteria of a ‘‘recommended’’ scale, or ‘‘listed’’ scales, which had little or no psychometric data to assess (Friedman et al., 2010). The Fatigue Assessment Inventory was suggested for both screening and severity. The Functional Assessment of Chronic Illness Therapy-Fatigue was recommended for screening and suggested for severity. The Multidimensional Fatigue Inventory was suggested for screening and recommended for severity. The Parkinson Fatigue Scale was recommended for screening and suggested for severity. The Fatigue Severity Inventory was listed for both screening and severity. The Fatigue Impact Scale for Daily Use was listed for screening and suggested for severity. Visual Analogue and Global Impression Scales were listed for screening and severity.

In terms of validity, the FSS discriminates significantly between healthy individuals and patients with fatigue-associated diseases, such as PD, multiple sclerosis, and postpoliomyelitis (Merkies et al., 1999; Vasconcelos et al., 2006; Armutlu et al., 2007). Furthermore, the FSS detects chronic fatigue syndrome (CFS) patients with 90% sensitivity and 84% specificity, which shows that results from the FSS are accurate indicators of clinicians’ diagnostic decisions regarding fatigue (Jason et al., 2011). With respect to reliability, the FSS demonstrates high internal consistency, with Cronbach’s alpha values that exceed 0.80 (Krupp et al., 1989; Taylor et al., 2000; Ziino and Ponsford, 2005; Armutlu et al., 2007). Although treatment of fatigue in PD continues to be a challenge, multiple pharmacological and non-pharmacological interventions, such as Doxepin, Rasagiline and exercise, are under investigation (Friedman et al., 2010; Elbers et al., 2016). Effective methods of measuring fatigue, such as the FSS, facilitate the detection as well as the eventual treatment of fatigue. The challenge in the clinic, though, is the wide array of potential non-motor symptoms and the need for their effective and rapid detection. Developing abbreviated versions of existing instruments could provide help while the selection of the most relevant features needs to be determined carefully.

Several statistical methods might be employed to select the FSS items that most accurately predict whether a patient will have clinically significant fatigue. A promising machine learning technique for this purpose is random forest, which has been applied by numerous clinical studies (Bukhari et al., 2016; Perrin et al., 2017; Mun and Geng, 2019; Byeon, 2020). Random forest appeared to be one of the most common algorithm in clinical studies for supervised classification and variable importance, hence it was judged to be a good starting point for our analyses. Random forest is a supervised classification algorithm (i.e. a statistical model that learns from the training data and classifies a new unseen test sample into one of multiple predefined categories) called “random forest” (Fawagreh et al., 2014). As a part of building its classification structure, a random forest calculates feature importance, which is a measure of how much impact a variable has in making the classification decisions (Fawagreh et al., 2014); if certain variables have higher feature importance scores, then these variables are more important predictors of classification.

However, critiques have pointed out that the random forest procedure can lead to unstable results in prediction and assessment of feature importance (Calle and Urrea, 2011; Wang et al., 2016). Feature importance scores generated by random forest can have problematic intrinsic stability, meaning that the scores are inconsistent across different iterations of the algorithm on the same dataset (Wang et al., 2016). While previous medical research using random forest may have led to valuable findings, it has generally relied on one iteration of the random forest to derive its conclusions and inform clinical decision making (Bukhari et al., 2016; Perrin et al., 2017; Yekkala et al., 2017; Chicco and Rovelli, 2019; Mun and Geng, 2019; Byeon, 2020). In fields outside of medicine, researchers adopted the practice of averaging feature importance scores across multiple iterations of random forest in order to better account for the possible fluctuations in results (Fox et al., 2017).

An additional analytical approach to help address the above weaknesses of random forest would be a modification of the random forest algorithm, called Boruta, which was used by Leong and Abdullah (2019) to determine the most important features in predicting Alzheimer’s disease. Boruta is a statistically grounded algorithm for automated feature selection (Kursa and Rudnicki, 2010). In addition to generating more robust feature importance scores compared to the unmodified random forest algorithm, Boruta can also help discriminate between relevant and irrelevant features for classification (Kursa and Rudnicki, 2010).

Another approach that might elucidate how the variance of the FSS dataset can be captured by FSS items is principal components analysis (PCA). PCA reduces the dimensionality of large datasets while minimizing information loss, hence making them easier to work with (Joliffe and Cadima, 2016). This is done by creating new uncorrelated variables while maximizing variance (i.e. useful statistical information), which act as summaries of the original variables (Joliffe and Cadima, 2016). These new variables are referred to as principal components (PCs), and PCs are numbered in the order of descending variance (i.e. PC1 has the largest variance, PC2 has the second largest variance, etc.) (Joliffe and Cadima, 2016). A medical application of PCA was demonstrated by Witteveen et al. (2017), where a high-dimensional dataset of inflammatory markers was simplified to three principal components, which facilitated data analysis.

In addition to statistical approaches, research has suggested that analyzing male and female PD patients separately may yield noteworthy findings. Biological sex appears to play a significant role in Parkinson’s disease, as various differences in the presentation of PD and its comorbidities for males vs. females have been reported, including aspects such as rapid eye movement sleep behavior disorder, verbal fluency, depression, dyskinesia and visuospatial function (Fernandez et al., 2000; Scott et al., 2000; Locascio et al., 2003; Baba et al., 2005; Ozekmekci et al., 2005; Accolla et al., 2007; Davidsdottir et al., 2008; Yoritaka et al., 2009). Furthermore, studies have shown the incidence of PD in men are 1.5 times higher than that of women (Wooten et al., 2004).

The primary aim of this study is to identify which items of the FSS best predict clinically significant fatigue in male and female PD patients comparing three different statistical analysis methods: random forest, Boruta and PCA. The secondary aim of this project is to discover additional statistical differences between males and females in the presentation of PD and its comorbidities, with the ultimate purpose of assisting clinicians to better recognize and treat these symptoms and comorbidities.

## Methods

### Data Collection

Two hundred and seventy-two participants with PD were enrolled through the Parkinson’s Research Centre (PPRC) at the University of British Columbia (UBC), Canada. All patients provided informed consent and the studies received research ethics approval. The following data was extracted: patient ID, visit number, disease status, sex, age, and FSS scores (items 1−9 and total score, with a total score of 36 or greater indicating clinically significant fatigue).

### Prediction Problem

This is a retrospective diagnostic study involving the categorical classification of patients into clinically significant fatigue or non-clinically significant fatigue using a clinical database of PD patients. The independent variables are each of the FSS items, and the dependent variable is clinically significant fatigue status.

### Preparation for Model Building

Preprocessing consisted of removing incomplete records, duplicate records and records of control patients. Furthermore, only records from the first clinic visit were used, since most patients did not have data associated with subsequent visits and thus the sample size appeared inadequate for analysis. The remaining dataset of 182 PD patients’ records contained no redundant independent variables with a predominant single value. Patient demographics of the pre-screening and post-screening datasets were displayed in the results section.

Quality metrics to assess the validity of the results were accuracy, sensitivity, specificity, positive predictive value, and negative predictive value, and these metrics are discussed in the results section.

### Building the Predictive Model

This project features three analytical approaches using random forest, Boruta and PCA. The methodology for each approach is discussed separately below. Furthermore, for transparency and reproducibility, the code used to perform the analyses can be found at https://github.com/dg2lee/Data-driven-prediction-of-PD.

### Methodology – Random Forest

Random forest is a supervised classification algorithm (i.e. a statistical model that learns from the training data and classifies a new unseen test sample into one of multiple predefined categories) (Fawagreh et al., 2014). As a part of building its classification structure, a random forest calculates feature importance, which is a measure of how much impact a variable has in making the classification decisions (Fawagreh et al., 2014); if certain variables have higher feature importance scores, then these variables are more important predictors of classification.

To train a random forest, the data is initially divided into a training set and a testing set. Then, the algorithm uses random subsets of the training set to build decision trees, which are flowcharts for deciding how to classify information (Reis et al., 2018). Decision trees, after having been generated using the training set’s features and data, can categorize given input data into the desired output categories. After the training phase is complete, random forest uses the created decision trees in order to predict the output categories of the data in the testing set (Breiman, 2001).

In this case, the dataset of 182 PD patients was randomly divided into a training (70%) and test (30%) set using the holdout method. The holdout method is a cross-validation approach used to assess the results of a classifier algorithm such as random forest. After partitioning the dataset into a training set and test set, the classifier is trained using only the training set, and then predicts the output values for the data in the testing set, which it has not yet seen (Koul et al., 2018). After partitioning, the training set was used to generate decision trees, which then predicted the classification of clinically significant vs. non-clinically significant fatigue for each patient in the testing set.

The RandomForestClassifier from the sklearn package running in Python 3.7 was used. Random stratified sampling was used to create training and test sets which were 70 and 30% of the original dataset, respectively (test_size = 0.3, stratify = y). 10,000 decision trees were created for every iteration of the algorithm (n_estimators). Changes were also made to allow for exact reproduction of results (fixed random state).

The algorithm performed 20,000 iterations, and the means and standard deviations for prediction accuracy, sensitivity, specificity, and feature importance scores were calculated. Prediction accuracy indicated the fraction of times that the algorithm correctly predicted whether a patient suffered from clinically significant fatigue. Sensitivity indicated the fraction of times that the algorithm correctly predicted disease status in patients with clinically significant fatigue. Specificity indicated the fraction of times that the algorithm correctly predicted non-disease status in patients without clinically significant fatigue. Feature importance scores indicated the impact of each FSS item in making the classification prediction.

### Methodology – Boruta

Boruta is a feature-selection algorithm run as a modification of the random forest algorithm. The Boruta algorithm computes average feature importance values based on numerous iterations of the random forest algorithm (100 by default) in order to increase the robustness of the feature importance results (Kursa and Rudnicki, 2010). For each iteration of random forest, Boruta generates shadow features, which are randomly mixed values copied from the original dataset. A variable is considered relevant for classification if its feature importance score is greater than that of the best shadow (randomly assigned) feature, and any variable that cannot satisfy this condition is deemed tentative or irrelevant.

Boruta was implemented in R version 4.0.0 using RStudio as the integrated development environment. Default parameters of the function Boruta were altered. In order to facilitate decision making about which variables are relevant or irrelevant for classification, the maximal number of importance source runs was increased to 500 (maxRuns = 500) and the function TentativeRoughFix was applied. Changes were also made to allow for exact reproduction of results for both functions [set.seed (1 … 10)].

The algorithm performed 20,000 iterations, and the means and standard deviations for feature importance scores were calculated. Unlike random forest, Boruta does not directly predict classification outcomes but rather assigns a value of importance for each feature, thus prediction accuracy was not reported. Feature importance scores indicated the impact of each FSS item in making the classification prediction.

### Methodology – PCA

PCA is defined as an orthogonal linear transformation that employs a scalar projection to transform the existing dataset to a new coordinate system, so that the greatest variance lies on the first coordinate (called the first principal component), the second greatest variance lies on the second coordinate (called the second principal component), and so on (Joliffe and Cadima, 2016).

One useful application of PCA is generating a loadings plot, which is a visualization of how strongly each feature or variable in the original dataset influences the first and second principal components. A loadings plot is a convenient method for holistically viewing the relationships between variables in a dataset: the vectors of positively correlated variables are close together and form acute angles, while uncorrelated variables’ vectors are close to orthogonal, and the vectors of negatively correlated variables form obtuse angles (Santos et al., 2019). In other words, strongly related variables will appear as clusters on the loadings plot (David and Jacobs, 2014).

PCA was implemented in IBM SPSS Statistics version 20. The Varimax rotation was used and loadings plots were generated.

## Results

### Preparation for Model Building

The initial data collection yielded 272 patients’ records, which included incomplete records, duplicate records and records of control patients. 37.1% of records were female, and the remainder were male. The mean age was 58.6 years.

After preprocessing, the remaining dataset consisted of 182 PD patients, and each patient record had 10 features: scores on FSS items 1–9 and total FSS score. 37.9% of patients were male, and the remainder were female. The mean age was 58.7 years. A total of 78 patients (48 males, 30 females) had clinically significant fatigue. This information is reported in Table 1-0.

**TABLE 1-0:** Pre-screening and post-screening patient demographics.

	Pre-screening	Post-screening
*n*	274	182
Age	64.90179	64.97802
%Female	0.370536	0.379121
PD disease duration (years)	6.325893	6.236264
Age of PD onset	58.57589	58.74176

### Results – Random Forest

#### Predictive Performance

**FIGURE 1:**
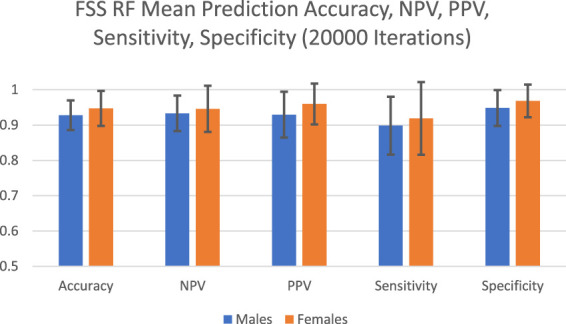
FSS random forest prediction accuracy, NPV, PPV, sensitivity, and specificity for 20,000 iterations.

The predictive performance of random forest in terms of accuracy, NPV, PPV, sensitivity and specificity are reported in Figure 1 and Table 1. The algorithm took into account all 9 FSS items and showed good and consistent overall predictive performance across 20,000 iterations. For males, the mean predictive accuracy, NPV, PPV, sensitivity, and specificity were 0.93 (SD = 0.042), 0.93 (SD = 0.050), 0.93 (SD = 0.065), 0.90 (SD = 0.082) and 0.95 (SD = 0.051), respectively. For females, the mean predictive accuracy, NPV, PPV, sensitivity, and specificity were 0.95 (SD = 0.065), 0.95 (SD = 0.065), 0.96 (SD = 0.058). 0.92 (SD = 0.103) and 0.97 (SD = 0.046), respectively.

**TABLE 1:** FSS male and female random forest prediction accuracy, NPV, PPV, sensitivity, and specificity for 20,000 iterations.

	**Males**					**Females**				
	Accuracy	NPV	PPV	Sensitivity	Specificity	Accuracy	NPV	PPV	Sensitivity	Specificity
Mean	0.92776	0.933181	0.929232	0.898279	0.948398	0.947052	0.945801	0.959659	0.918789	0.96825
SD	0.041793	0.050311	0.064836	0.081978	0.05066	0.049742	0.065439	0.057706	0.102778	0.046305

#### Feature Importance Scores

**FIGURE 2:**
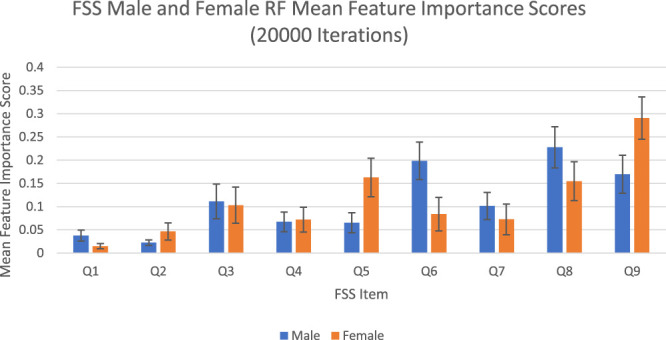
FSS male and female random forest mean feature importance scores and standard deviations for 20,000 iterations. Error bars represent 1 SD.

**FIGURE 3:**
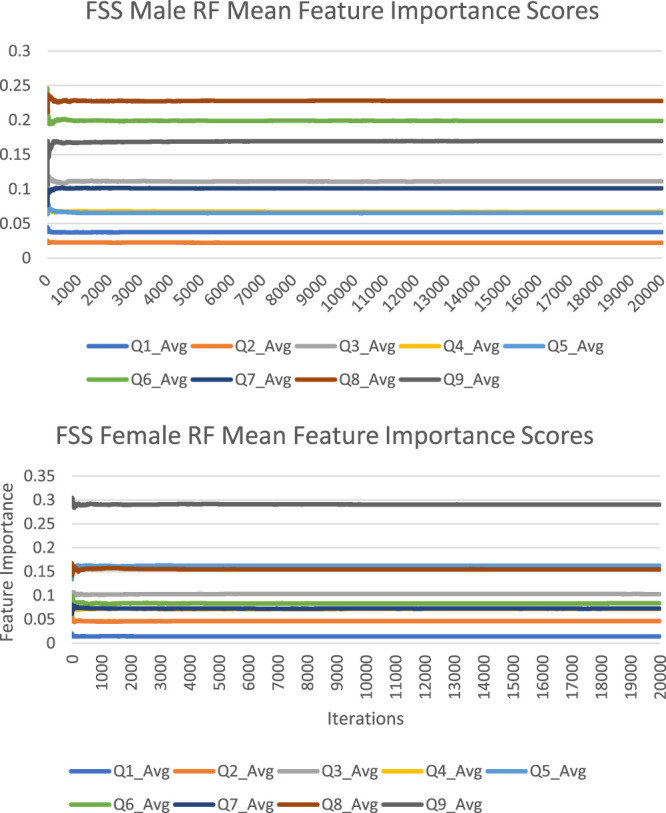
FSS male and female random forest mean feature importance score trends.

The feature importance scores generated by random forest for each FSS item are reported in Figure 2 and Table 2. For both male and female PD patients, Q8 and Q9 were among the three most important predictors and Q1 and Q2 were among the least important predictors. Interestingly, Q5 was an important predictor for females, but Q6 was an important predictor for males. The mean feature importance score trends for each FSS item are shown by Figure 3 and confirm that these findings are stable beyond the 1000th iteration.

**TABLE 2:** FSS male and female random forest mean feature importance scores for 20,000 iterations.

	FSS male RF			FSS female RF		
Item	Mean Feature Importance Score (20,000 Iterations)	Standard Deviation	Relative Standard Deviation (%)	Mean Feature Importance Score (20,000 Iterations)	Standard Deviation	Relative Standard Deviation (%)
Q1	0.03761	0.01193	31.72358	0.01467	0.00555	37.83603
Q2	0.02230	0.00605	27.12389	0.04641	0.01855	39.96235
Q3	0.11110	0.03748	33.73266	0.10312	0.03891	37.72974
Q4	0.06701	0.02102	31.37262	0.07185	0.02672	37.19138
Q5	0.06515	0.02164	33.21931	0.16261	0.04137	25.44369
Q6	0.19859	0.04044	20.36445	0.08359	0.03609	43.17085
Q7	0.10116	0.02922	28.88751	0.07248	0.03308	45.64427
Q8	0.22752	0.04434	19.48748	0.15471	0.04199	27.13994
Q9	0.16956	0.04081	24.06811	0.29056	0.04552	15.66794

The instability of feature importance scores generated by random forest was demonstrated by relative standard deviation (RSD) values in Table 2. An FSS item’s RSD is the proportion of its standard deviation to the mean value, expressed as a percentage (SD/Mean Feature Importance Score*100%). RSD values ranged from 16 to 46%, with an average of 31%. This reflected the magnitude of instability of feature importance scores and feature importance rankings across different iterations.

### Results – Boruta

#### Feature Importance Scores

**FIGURE 4:**
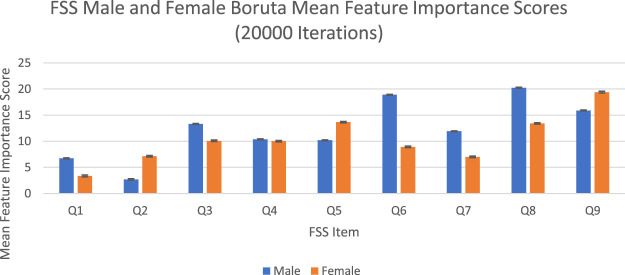
FSS male and female Boruta mean feature importance scores for 20,000 iterations. Error bars represent 1 SD.

**FIGURE 5:**
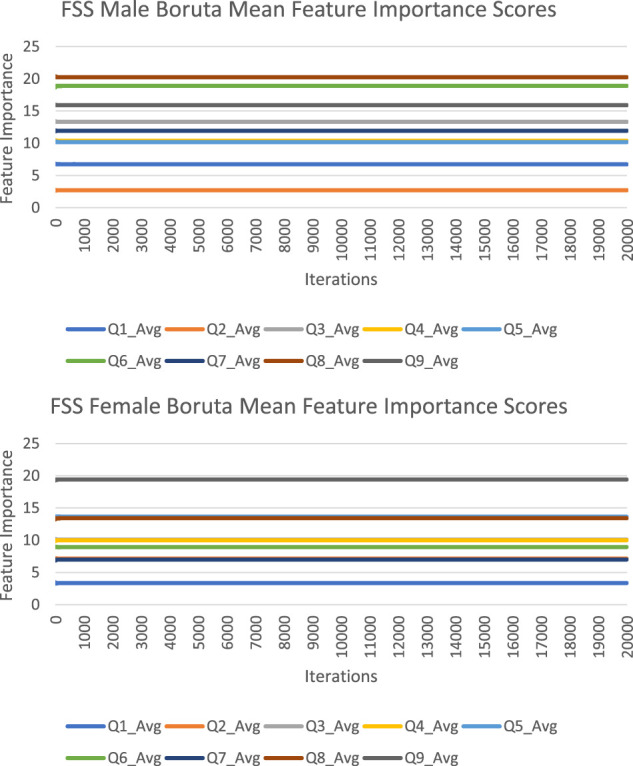
FSS male and female Boruta mean feature importance score trends.

The feature importance scores generated by Boruta for each FSS item are reported in Figure 4 and Table 3. The results were nearly identical to those obtained from random forest. For both male and female PD patients, Q8 and Q9 were among the three most important predictors and Q1 and Q2 were among the least important predictors. Furthermore, Q5 was an important predictor for females, but Q6 was an important predictor for males. All items were deemed to be relevant for classification. The mean feature importance score trends for each FSS item are shown by Figure 5 and confirm that these findings are stable beyond the 1000th iteration.

**TABLE 3:** FSS male and female Boruta mean feature importance scores for 20,000 iterations.

	FSS male Boruta			FSS female Boruta		
Item	Mean Feature Importance Score (20,000 Iterations)	Standard Deviation	Relative Standard Deviation (%)	Mean Feature Importance Score (20,000 Iterations)	Standard Deviation	Relative Standard Deviation (%)
Q1	6.73852	0.09649	1.43192	3.35856	0.18180	5.41291
Q2	2.70242	0.12120	4.48495	7.13703	0.15366	2.15294
Q3	13.32298	0.07694	0.57750	10.09510	0.13804	1.36735
Q4	10.37314	0.07883	0.75998	10.02586	0.13516	1.34812
Q5	10.19065	0.08300	0.81444	13.65371	0.13858	1.01499
Q6	18.89240	0.08789	0.46524	8.92959	0.14322	1.60390
Q7	11.92685	0.07753	0.65009	7.01361	0.15484	2.20775
Q8	20.24304	0.09252	0.45704	13.42091	0.14033	1.04563
Q9	15.90572	0.08266	0.51966	19.41678	0.16477	0.84861

Boruta clearly outperformed random forest with respect to stability of feature importance scores. RSD values ranged from 0 to 5%, with an average of 2%. In contrast to random forest, Boruta has robust feature importance scores and feature importance rankings across different iterations.

### Results – PCA

#### Loadings Plot

**FIGURE 6:**
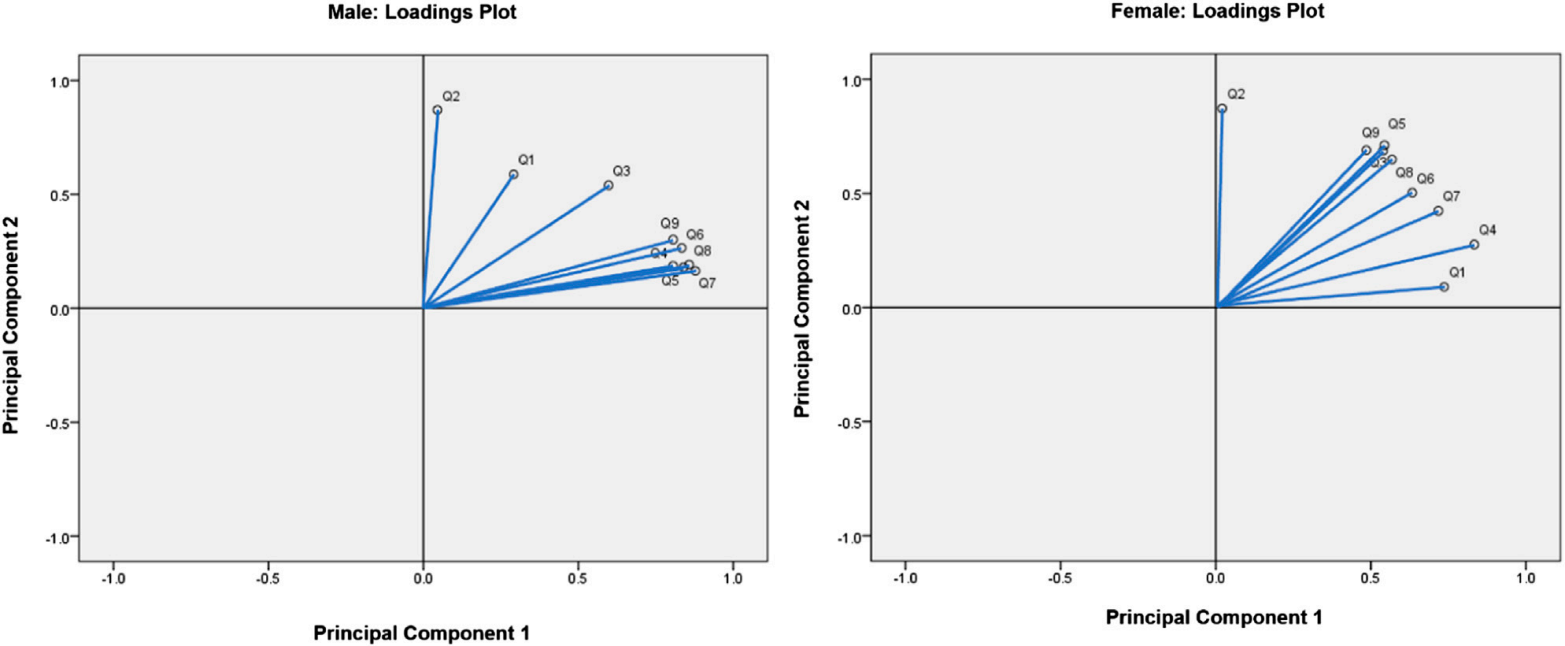
Loadings plots for males and females.

The loadings plot generated a visualization of the relationships between FSS items. Strongly related variables appear as clusters on the loadings plot since the vectors of positively correlated FSS items form acute angles. In contrast, uncorrelated variables’ vectors are approximately perpendicular, and the vectors of negatively correlated variables form obtuse angles. For both males and females, it was apparent that Q1 and Q2 were the furthest apart from the cluster formed by other items, meaning that Q1 and Q2 were the least related to other questions (Figure 6).

## Discussion

While PD is widely known as a movement disorder, a challenge in the clinic about seeing PD patients is the wide variety of potential non-motor symptoms, such as fatigue, and the need for the effective and rapid detection of these symptoms. PD patients can come with many non-motor symptoms and there is a need to prioritize which symptoms should be examined more closely.

Currently, to measure these non-motor symptoms, patients are given numerous questionnaires which can be time consuming and burdensome. However, many of these questionnaires, including the Fatigue Severity Scale, are not a mandatory part of assessing a Parkinson’s disease patient.

The aim of this study was to improve the clinical detection of fatigue in PD by determining which items of the FSS best predict clinically significant fatigue. These questions could be quickly asked in a patient interaction to help decide whether to further investigate a PD patient’s fatigue. Furthermore, these questions may used to make condensed versions of existing questionnaires. Additionally, the findings of this project may support the development of new treatments for fatigue in PD by facilitating the creation of abbreviated versions of existing instruments such as the FSS. Abbreviated questionnaires may be quicker to administer and be less burdensome to patients, so this could allow clinicians to use these tools more frequently. Furthermore, increased frequency of administration may bolster data collection for research, which may facilitate the development of new treatments. This is particularly significant at a time more research is required regarding treatments; there is no currently agreed upon gold standard treatment of fatigue in PD, and multiple pharmacological and non-pharmacological interventions, such as Doxepin, Rasagiline and exercise, are under investigation (Friedman et al., 2010; Elbers et al., 2016).

To achieve this purpose, random forest, which has been applied by clinical studies to determine the most important predictors of various disorders such as mild cognitive impairment and depression, was applied on the FSS dataset (Perrin et al., 2017; Byeon, 2020). Past studies used predictive accuracy as a performance metric to evaluate the credibility of the generated feature importance scores (Fox et al., 2017). Past medical applications of random forest demonstrated a wide range of prediction accuracy values, from 0.25 to 0.925 (Bukhari et al., 2016; Perrin et al., 2017; Yekkala et al., 2017; Mun and Geng, 2019; Byeon, 2020). In the current study, the algorithm showed high overall predictive performance across 20,000 iterations (Figure 1). For males, the mean predictive accuracy, NPV, PPV, sensitivity, and specificity were 0.93 (SD = 0.042), 0.93 (SD = 0.050), 0.93 (SD = 0.065), 0.90 (SD = 0.082) and 0.95 (SD = 0.051), respectively. For females, the mean predictive accuracy, NPV, PPV, sensitivity, and specificity were 0.95 (SD = 0.065), 0.95 (SD = 0.065), 0.96 (SD = 0.058). 0.92 (SD = 0.103) and 0.97 (SD = 0.046), respectively. These results support the legitimacy of the generated feature importance scores for each FSS item.

However, feature importance scores generated by random forest are inherently unstable (Calle and Urrea, 2011; Wang et al., 2016). To better account for the possible fluctuations in results, feature importance scores were averaged across 20,000 iterations of random forest. In addition, this study measured the standard deviation of feature importance scores as a measure of stability. Furthermore, Boruta, a modification of the random forest algorithm, was reported to generate more robust feature importance scores compared to random forest, as well as to discriminate relevant and irrelevant features for classification (Kursa and Rudnicki, 2010).

The averaged feature importance scores generated by random forest for each FSS item were reported in Figure 2, while Boruta’s feature importance scores were reported in Figure 4. The results obtained from random forest and Boruta were nearly identical. For both male and female PD patients, Q8 (“Fatigue is among my three most disabling symptoms”) and Q9 (“Fatigue interferes with my work, family or social life”) were among the most important predictors and Q1 (“My motivation is lower when I am fatigued”) and Q2 (“Exercise brings on my fatigue”) were among the least important predictors. Interestingly, Q5 (“Fatigue causes frequent problems for me”) was an important predictor for females, but Q6 (“My fatigue prevents sustained physical functioning”) was an important predictor for males. These findings suggest that, in the clinic, assessing how disabling fatigue is compared to other symptoms and interference with work, family and friends may be the most informative. Furthermore, it may be most helpful for clinicians to assess frequency of fatigue-related complaints in females and endurance-related complaints in males.

Feature importance scores’ SD were very large for RF and extremely small for Boruta. The instability of an FSS item’s feature importance score was shown by the relative standard deviation (RSD = SD/Mean Feature Importance Score*100%). For random forest, RSD values ranged from 14 to 66%, with an average of 35%. In contrast, Boruta clearly outperformed the unmodified random forest with respect to stability of feature importance scores, with RSD values ranged from 0 to 5%, with an average of 1%, which was expected since the Boruta algorithm utilized average feature importance values based on numerous iterations of the random forest algorithm (maximum 500) for more robust results. These results suggest that it is possible to compensate for the inherent instability of random forest by running multiple iterations, using mean scores, and reporting SD values. Furthermore, future researchers are encouraged to consider supplementing random forests with Boruta, or other feature-selection algorithms, which yielded much more stable results. Considering that previous medical research has relied on findings from one iteration of the random forest, adopting these suggested changes may lead to improved clinical decision-making recommendations (Bukhari et al., 2016; Perrin et al., 2017; Yekkala et al., 2017; Chicco and Rovelli, 2019; Mun and Geng, 2019; Byeon, 2020).

PCA, which is generally used to reduce a dataset’s dimensionality, was applied in this study in order to holistically view the relationships between FSS items (Witteveen et al., 2017). The PCA loadings plot visualized the relationships between variables in a dataset, grouping highly related FSS items together (David and Jacobs, 2014). These groups can then be compared to the results of the random forest and Boruta algorithms, consisting of groups of FSS items which are the strongest predictors of clinical fatigue. If the groupings generated by PCA, random forest and Boruta align, the strongest predictors of clinically significant fatigue in the FSS would be clearly supported by three data-driven approaches. For both males and females, it was apparent that Q1 (“My motivation is lower when I am fatigued”) and Q2 (“Exercise brings on my fatigue”) were the furthest apart from the cluster formed by other items, meaning that Q1 and Q2 were the least related to other questions (Figure 6). This supported the results obtained from both the random forest and Boruta algorithms, which revealed that Q1 and Q2 were among the least important predictors of clinically significant fatigue.

### Limitations

This study used the random forest and Boruta algorithms for data analysis, and these findings may not be replicable with other machine learning algorithms. Distinct algorithms have contrasting methods of identifying the most important predictors in a dataset, and thus place variable amounts of emphasis on what defines an important predictor.

Additionally, the results obtained from this study may not be generalizable to the general population of PD patients. With respect to sample size, there have been clinical papers using random forest that used a wide range of participant numbers, both less and greater than our sample size of 182. For example, Bukhari et al. (2016), Perrin et al. (2017), Yekkala et al. (2017), Mun and Geng (2019), and Byeon (2020) used sample sizes of 37, 270, 307, 212 and 96, respectively. Nevertheless, a smaller sample size decreases the external validity of the findings in this study in comparison with studies with a greater sample size.

Further research is required to replicate these findings with other machine learning algorithms, and using a larger and more representative sample of PD patients.

## Conclusion

Random forest, Boruta and PCA results converged to demonstrate that Q8 (“Fatigue is among my three most disabling symptoms”) and Q9 (“Fatigue interferes with my work, family or social life”) may be among the most important predictors and Q1 (“My motivation is lower when I am fatigued”) and Q2 (“Exercise brings on my fatigue”) may be among the least important predictors. Q5 (“Fatigue causes frequent problems for me”) may be an important predictor for females, and Q6 (“My fatigue prevents sustained physical functioning”) may be an important predictor for males. Therefore, when assessing fatigue in PD patients, it may be most informative for clinicians to assess how disabling fatigue is compared to other symptoms and interference with work, family and friends. Furthermore, it may be most helpful for clinicians to assess frequency of fatigue-related complaints in females and endurance-related complaints in males. Although the same conclusion was reached across the three data-driven approaches, it may be beneficial to consider supplementing random forests with Boruta, which yielded much more stable results. Adopting these suggested changes may lead to improved clinical decision-making recommendations. However, further research in this area would be beneficial in order to replicate these findings with other machine learning algorithms, and using a larger and more representative sample of PD patients.

## Data Availability

The data analyzed in this study is subject to the following licenses/restrictions: The dataset is not publicly available since it may contain information that could compromise research participants’ privacy/consent. Requests to access these datasets should be directed to Dong Goo Lee, dgl@alumni.ubc.ca.
